# Totally 3D Endoscopic Repair of Double-Chambered Right Ventricle

**DOI:** 10.1016/j.atssr.2022.09.003

**Published:** 2022-09-15

**Authors:** Riku Kato, Soh Hosoba, Daichi Fukumi, Toshiaki Ito

**Affiliations:** 1 Department of Cardiovascular Surgery, Japanese Red Cross Aichi Medical Center Nagoya Daiichi Hospital, Michishita, Japan; 2 Department of Pediatrics, Japanese Red Cross Aichi Medical Center Nagoya Daiichi Hospital, Michishita, Japan

## Abstract

Double-chambered right ventricle (DCRV) is a rare malformation that may develop after ventricular septal defect (VSD) closure. We describe a case of a 34-year-old women who underwent VSD closure in her infancy with a small Y-collar incision. She developed dyspnea on exertion and was recommended for surgery. She desired minimally invasive surgery for a cosmetic reason. We performed a totally 3e-dimensional endoscopic minimally invasive repair of DCRV. Endoscopic repair of DCRV after VSD closure for an adult is possible with an excellent view. We describe our approach, which has overcome unique technical hurdles and has yielded favorable outcomes.

Double-chambered right ventricle (DCRV) is a form of congenital heart disease. In DCRV, there is a mid-ventricular obstruction that divides the right ventricle into a high-pressure proximal portion and a low-pressure distal portion. DCRV presents in 1% of all patients with congenital heart disease. It has been reported that 70%-80% of DCRV patients are accompanied by a ventricular septal defect (VSD).^1^ Symptomatic DCRV may require surgical intervention. We routinely perform minimally invasive cardiac surgery (MICS) for valves or simple congenital defects, such as atrial septal defect and VSD. Given the experience of VSD repair with a 3-dimensional (3D) endoscope, we deemed totally endoscopic repair for DCRV feasible. Herein, we report a case of totally 3D-endoscopic repair of DCRV.

A 34-year-old woman who underwent VSD closure as a 1-year-old developed an intraright ventricular pressure gradient. She was diagnosed with DCRV at age 25 years and was referred for surgical evaluation, at which time she was asymptomatic and declined surgery. She strongly desired minimally invasive cardiac surgery for a cosmetic reason, despite her existing Y-collar incision from the prior sternotomy. Nine years later, a transthoracic echocardiogram showed moderate tricuspid regurgitation and mid-ventricular obstruction with an intraright ventricular pressure gradient. Her right heart catheterization showed that the right ventricular pressure was 90 mm Hg and the pressure gradient at the right ventricular outflow was 64.5 mm Hg. We deemed her a candidate for a totally endoscopic DCRV repair.

She was placed in a 30° left lateral decubitus position with the right arm fixed over the head. Cardiopulmonary bypass was established through the right groin. A 10-mm trocar for a 3D endoscope (Karl Storz, Tuttlingen, Germany) was inserted through the fifth intercostal space in the right mid-axillary line. The main 3-cm incision was made at the sixth intercostal space without rib-spreading. A 1.5-cm incision for a lefthand instrument was placed at the third intercostal space (Figure 1).^2^ After snaring the superior vena cava and the inferior vena cava, the ascending aorta was cross-clamped, and cardiac arrest was achieved through antegrade cardioplegia. Through a right atrial incision, the prior VSD repair site and abnormal muscular bands were identified (Figure 2A). The abnormal muscular bands at the inferior wall and in the right ventricular outflow tract were resected. These bands were excised with scissors and cut-set-cautery through the tricuspid valve (Figure 2B). Subsequently, the tricuspid ring annuloplasty was performed. The right atrium was closed, and the patient was weaned off cardiopulmonary bypass (Video). Transesophageal echocardiogram demonstrated no accelerating flow in the right ventricular outflow tract (Figure 3). Aortic cross-clamp, cardiopulmonary bypass, and operation times were 61, 157, and 202 minutes, respectively. The patient was extubated 4 hours after the operation. She was transferred to the floor the next day. She had an uneventful recovery and went home on postoperative day 6. Her echocardiogram showed normal right ventricular function and no tricuspid regurgitation. She remained asymptomatic at 18-month follow-up.

**Figure 1:**
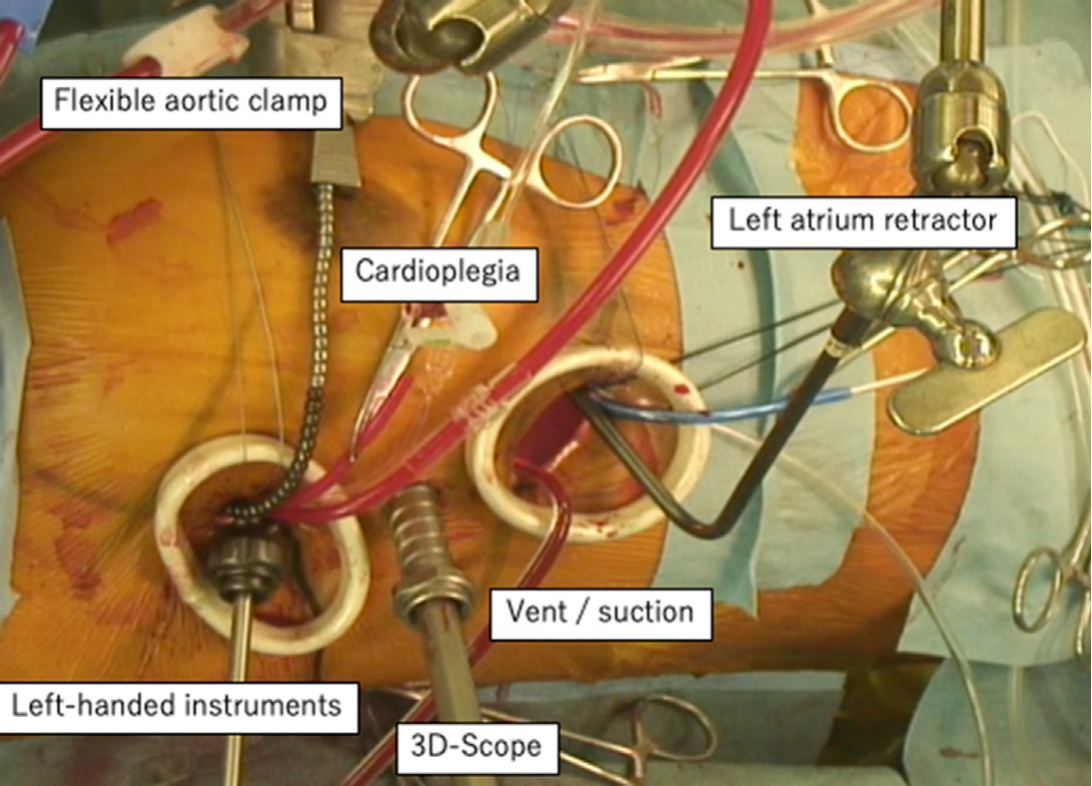
Setup of the “2-window” technique.

**Figure 2:**
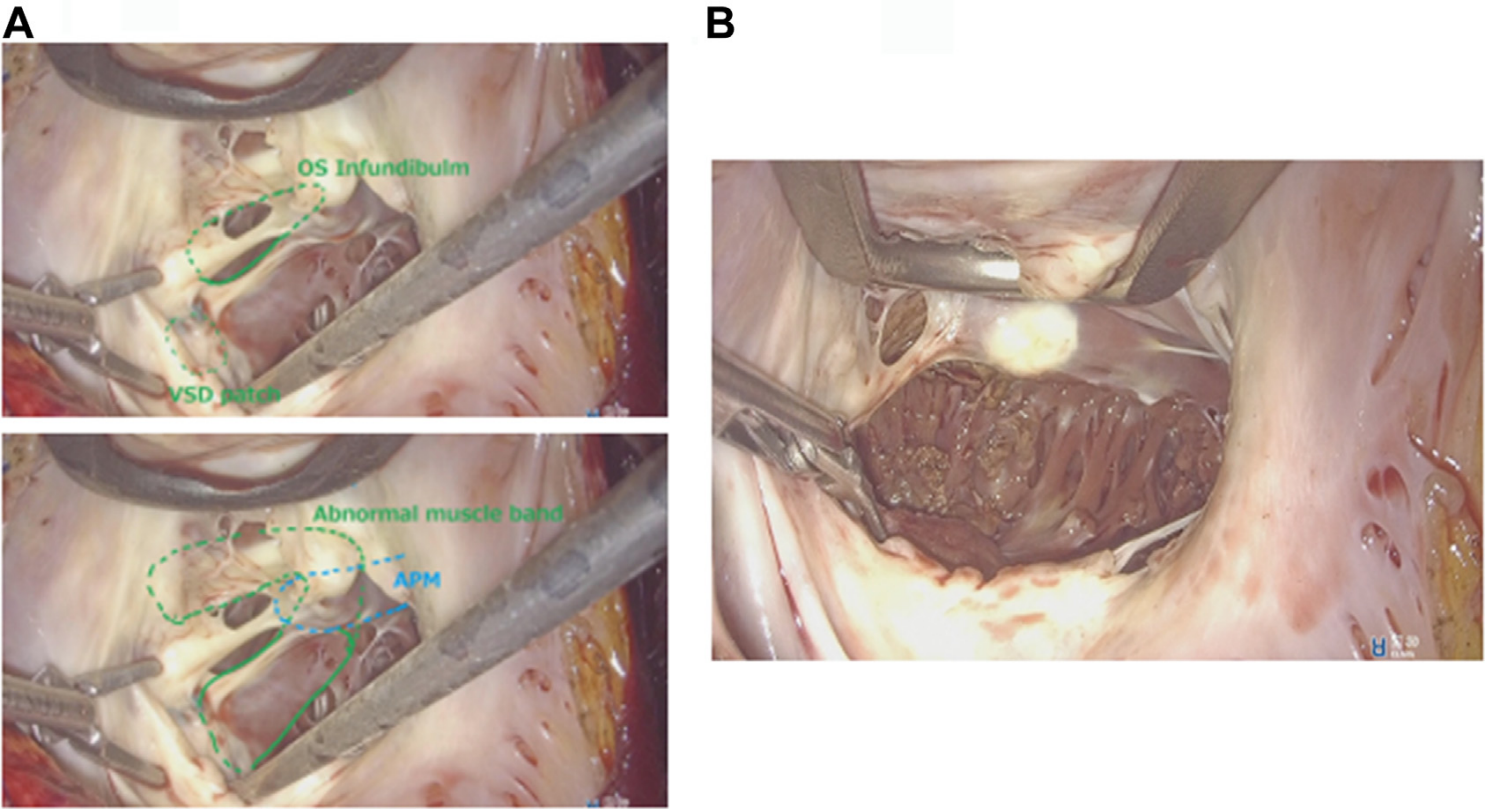
Three-dimensional endoscopic view of the double-chambered right ventricle before resection (A), and after resection (B). (APM, anterior papillary muscle; VSD, ventricular septal defect.)

**Figure 3:**
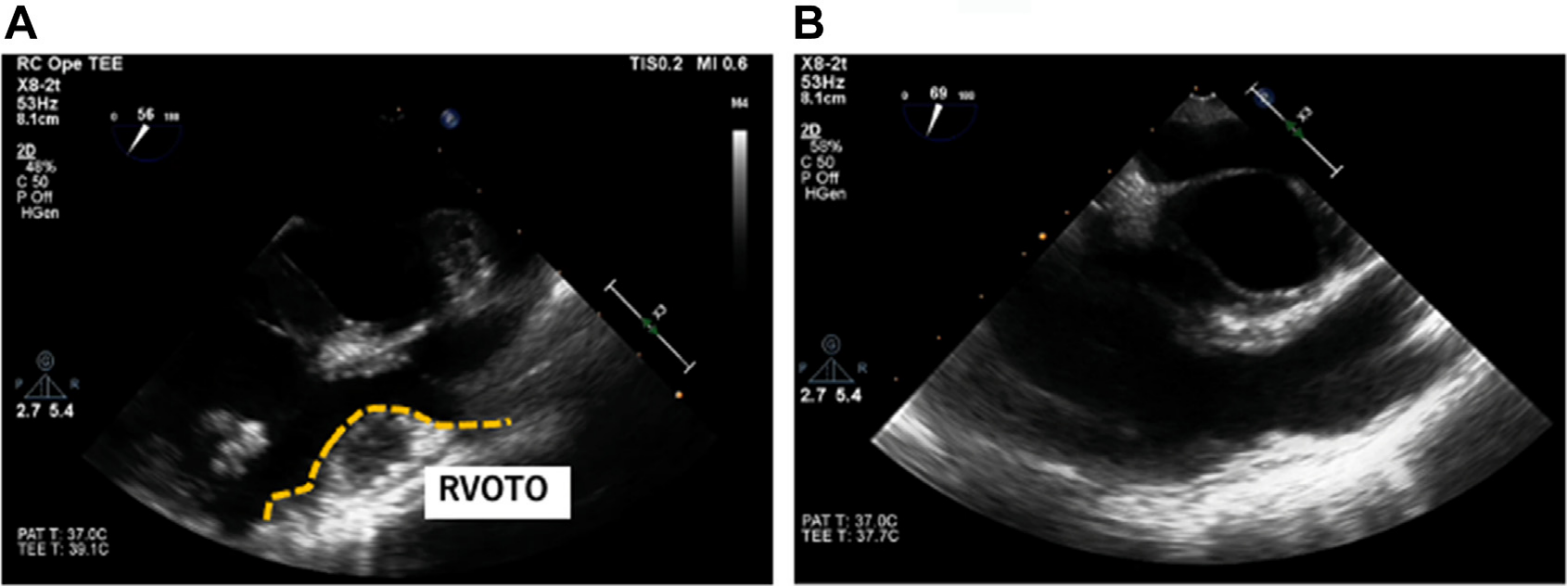
Transesophageal echocardiogram before operation (A), and after operation (B). (RVOTO, right ventricular outflow tract obstruction.)

## Comment

DCRV is a rare congenital heart disease in which the right ventricle is divided into two chambers by an abnormal muscular band. According to the American Heart Association /American College of Cardiology guideline, surgical intervention is indicated at intraright ventricular pressure peak gradient above 64 mm Hg.^3^ Our patient was symptomatic with an intraright ventricle pressure peak gradient of 64.5 mm Hg. The surgical outcome for DCRV is generally good, as Said and associates^4^ reported excellent functional and hemodynamic results in the long term after surgical correction of DCRV.

The optimal approach for the resection of the obstructing abnormal muscular band remains controversial. Options for the approach include transventricular, transatrial, combined transatrial-pulmonary and combined transatrial-transventricular approaches.^4^^,^^5^ The transventricular approach offers an appropriate surgical view to identify the abnormal muscular band. On the other hand, this approach may reduce the right ventricular function and may cause ventricular arrhythmia after the right ventriculotomy. At our institution, we perform VSD patch closure routinely via a transatrial approach using the MICS approach with a 3D endoscope. From the preoperative assessment, we concluded that for this patient the totally endoscopic approach is feasible to approach the inferior muscular band and right ventricular outflow tract. In this case, our approach offered a good surgical view to correct DCRV without a right ventricular incision.

MICS after median sternotomy has better cosmetics and also safety benefits. In redo cases after a median sternotomy, the MICS approach can be performed safely, except in cases of severe adhesion in the right thoracic cavity.^6^ Some patients diagnosed with DCRV as adults have undergone median sternotomy as a child for VSD or tetralogy of Fallot. The lesser extent of dissection required is also an advantage of the MICS approach.

The totally 3D endoscopic minimally invasive repair of DCRV after VSD closure in an adult is possible with an excellent surgical view.
